# Our journey to digital curation of the Jeghers Medical Index

**DOI:** 10.5195/jmla.2017.47

**Published:** 2017-07-01

**Authors:** Lori Gawdyda, Kimbroe Carter, Mark Willson, Denise Bedford

## Abstract

**Background:**

Harold Jeghers, a well-known medical educator of the twentieth century, maintained a print collection of about one million medical articles from the late 1800s to the 1990s. This case study discusses how a print collection of these articles was transformed to a digital database.

**Case Presentation:**

Staff in the Jeghers Medical Index, St. Elizabeth Youngstown Hospital, converted paper articles to Adobe portable document format (PDF)/A-1a files. Optical character recognition was used to obtain searchable text. The data were then incorporated into a specialized database. Lastly, articles were matched to PubMed bibliographic metadata through automation and human review. An online database of the collection was ultimately created. The collection was made part of a discovery search service, and semantic technologies have been explored as a method of creating access points.

**Conclusions:**

This case study shows how a small medical library made medical writings of the nineteenth and twentieth centuries available in electronic format for historic or semantic research, highlighting the efficiencies of contemporary information technology.

## BACKGROUND

Harold Jeghers was a well-known medical educator of the twentieth century who contributed to defining Peutz-Jeghers syndrome [1, 2]. During his career, he collected approximately one million medical articles dating from the late 1800s to the 1990s. The collection followed him to several academic centers and teaching hospitals, and it eventually came to Youngstown, Ohio, in 1982. In his later years, Jeghers joined the faculty of the Internal Medicine Department at Northeast Ohio Medical University (NEOMED) and medical staff at St. Elizabeth Youngstown Hospital (SEYH), a community teaching hospital affiliated with NEOMED. His collection continued to be a teaching resource at SEYH and became known as the Jeghers Medical Index (JMI). The JMI has been self-supporting through a trust shortly after its purchase by the Department of Education at SEYH. An advisory board of physicians, hospital administrators, and community professionals has directed the activities of the JMI, aligning goals to the trust’s directive, and has safeguarded the collection for three decades.

Throughout the 1980s and early 1990s, Jeghers oversaw the continued accumulation of the collection, with articles from about seventy academic clinical journals. Internal medicine and surgery residents as well as medical students rotated through the JMI for one month. They were assigned a clinical problem to research, created an annotated bibliography, and presented their findings at the end of their rotations. These residents and students, assisted by JMI staff, learned the skills of finding medical literature before the widespread use of personal computers. In the early 1990s, the JMI was used less frequently in residence training; however, the medical staff continued to use the collection as a source of background information for lectures, poster presentations, and manuscript preparation.

By the late 1990s, the collection had grown to fill more than 165 fireproof cabinets holding 45,000 folders containing approximately 1 million medical articles consisting of 5 million pages. Initially, Jeghers used an anatomic scheme to categorize articles, but by the 1990s, the filing system had features of the Medical Subject Headings (MeSH) scheme used by the US

National Library of Medicine. As the collection grew, Jeghers’ indexing limited the efficiency of article retrieval. Additionally, notes written on the pages by patrons and indexers, along with paper deterioration, started to limit legibility of the articles.

## STUDY PURPOSE

A solution for dealing with the paper deterioration, legibility, retrieval efficiency, and continued relevance of the JMI in medical teaching required a modern approach. The authors planned to store the text of the articles in a logically organized, searchable digital database system, which would enable standards-based bibliographic metadata searches in both specific fields as well as the text. Herein, we describe the two-decade journey of converting a large paper collection to a specialized digital database.

## CASE PRESENTATION

Electronic archiving of the collection involved the Preservation Project, followed by the eJeghers Content Project. The Preservation Project included about 120,000 articles that predated 1965, which were chosen due to their deterioration. The collection contained copyrighted work subject to allowances permitting reproductions for preservation purposes. Although JMI staff consisted of only two full-time workers—a librarian and an assistant—digitization of the paper collection began in the late 1990s with little forethought that these efforts could last for decades.

Articles were scanned to tagged image file format (TIFF). A Microsoft SQL database was created, and bibliographic data were manually entered over two years with the assistance of five part-time students from the regional university. Concern about preserving the remaining print collection mounted as experience was gained in working with the pre-1965 articles. The brittleness of the paper sometimes slowed progress. After completing the Preservation Project, the advisory board decided to digitize the remaining collection due to paper deterioration and safety inspection concerns related to an emergency exit that was impeded by the filing cabinets. This part of the collection also contained copyrighted work, but the deteriorating condition of the material was considered to be a copyright exception that permitted reproduction for preservation. A detailed project management document was created to guide tasks, timelines, and milestones.

Subsequently, the eJeghers Content Project included digitizing the remaining 880,000 print articles, merging them with the digitized data of the Preservation Project, and integrating the collection into a flexible data management system specialized for storage and searching. Since inception of the first project, noticeable improvements evolved in scanning, optical character recognition (OCR) processing, and archival document formats. Experience gained in the first project showed the importance of using OCR software that met the project specifications. OCR software ABBYY Recognition Server, ABBYY Finereader Corporate, Cvision Maestro, Expervision Typereader, and AnyDoc were compared for speed, accuracy, and pricing. The price of purchasing software and hardware was compared to that of using a third-party vendor. ABBYY Recognition Server best met the project’s criteria. Similarly, data management vendors Google, GoPubmed, Exalead, Thunderstone, Greenstone, and dSpace were compared for cost, relevance, user interface, methodology, location of corporate headquarters, and willingness to customize software. Thunderstone and its TEXIS product most suitably met the project’s data management needs.

In developing the eJeghers Content Project, PubMed XML citations and a table of anatomic terms that Jeghers created to use in retrieving articles from the original collection were added to a specialized relational database, TEXIS. TEXIS is an SQL relational database with powerful text searching capabilities with its own scripting language, Vortex [3, 4]. PubMed records were loaded into TEXIS, while maintaining PubMed’s field structure.

Several computational techniques were used in extracting the needed bibliographic data from each article. These included rules using the position of alphanumeric characters and publication symbols. For example, a number following “Volume” was interpreted as the publication volume number and a number following “#” was interpreted as an issue number. Page numbers were considered more likely when at the top or bottom of a page than in the body of the text. Another important rule that was applied was clustering author names on the first page of an article.

**Figure 1:**
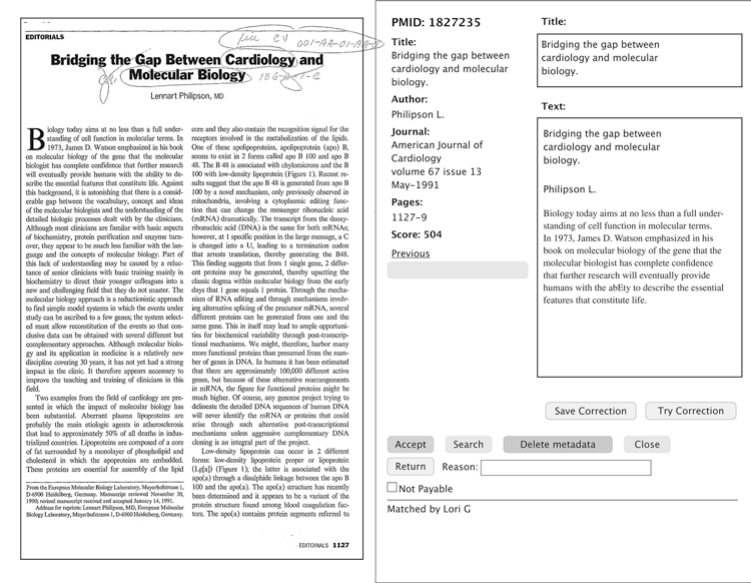
Reviewer interface for determining PubMed match accuracy

At the time of the project, PubMed citations did not exist for many articles published before 1960. An algorithm assigned a score that predicted if a PubMed citation matched a JMI article. Some articles required human review, based on the value of the match score. A user-friendly interface was developed to facilitate this review. Features of the interface are shown in Figure 1. The staff compared an article on the left-hand side of the screen with the algorithm-assigned PubMed record on the right-hand side. The reviewer had the option of accepting the record, searching for the correct PubMed record, or adding bibliographic data. Six people spent more than 2 years reviewing approximately 300,000 articles. At the project’s end, more than 900,000 articles had bibliographic metadata.

**Figure 2:**
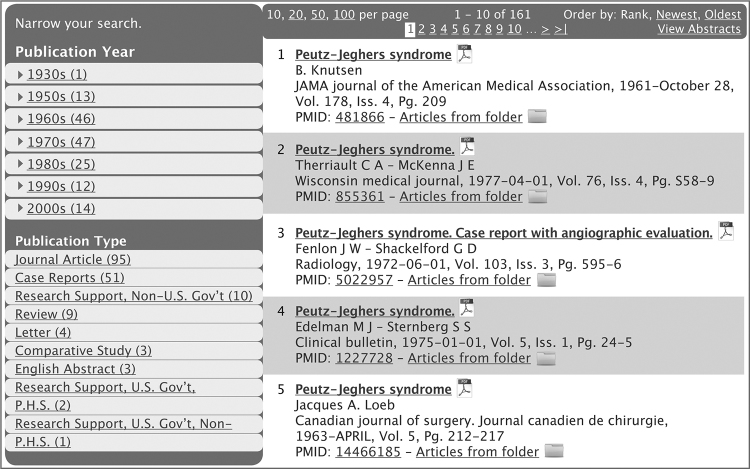
Display of query results

The completed TEXIS system manages about one million articles, comprising five million pages and containing over two billion words. The database, which is hosted online, offers three search options to assist in article retrieval. One search approach uses Boolean logic, with search and retrieval behaviors similar to PubMed. Users can query author, title, journal, abstract, and MeSH. A second approach—proximity search—uses the occurrences of terms and location of words in sentences, paragraphs, pages, or documents to retrieve articles. This approach takes advantage of the relationships between words and the clustering of words with concepts. Proximity search can extract concepts contained in the body of the articles. A third approach uses the indexing method that Jeghers developed that relies on anatomic and disease concepts and retrieves folders containing the article. Jeghers’ method is influenced by disease, organ system, physiology, and anatomy [5]. The public view of the database displays results initially by relevance. The display of results from a query is shown in Figure 2, with several options available for organizing the bibliographic data. Users can choose to list results by year of publication, with or without abstracts, or by study type. A fair use copy may be requested from the JMI librarian in compliance with copyright law.

## DISCUSSION

History plays an important role in anchoring the perspectives of researchers and practitioners alike in approaching medicine. In the context of medical advancements, knowledge of past questions and their bygone solutions are important in understanding the medical choices of today. Medicine and the concepts of disease are not fixed, as they change in response to social and environmental conditions and are understood at any time in strikingly different ways. The JMI now is a window into the past.

Although it is no longer directly involved in a teaching program, the JMI continues to be a source of articles and information for scholarly purposes, providing background to current questions. In a review of recent publications by SEYH staff, about 16% of publications referenced medical literature that was more than 20 years old. Many of these references came from the JMI collection. Use of the JMI is not restricted to SEYH staff. About 5% of interlibrary loan requests to the SEYH Medical Library can be found in the collection. Increased usage of the collection has been the result of inclusion in a discovery search service of the SEYH Medical Library. Over the past year, more than 2,000 hits or searches have been recorded.

Techniques to improve recall and accuracy of queries in the collection are now under consideration. Shifts in health care terms, meanings, and concepts over the years sometimes limit keyword queries that use current natural language expressions. Retrieval accuracy could be improved using semantic methods such as entity extraction and classification to create additional metadata from text in the collection. The metadata must be tagged to make it available for search. With such a large collection, meeting these requirements manually, with indexers and historians, is impractical [6].

Therefore, a pilot study was undertaken to investigate the automated gathering of supplemental metadata through entity extraction and classification using Reuter’s Open Calais [7] and SAS Content Categorization [8]. These methods rely on authority lists and rule sets used at the World Bank [9]. Terms and concepts of disease, body parts, symptoms, and treatments are drawn from Jeghers’ indexing scheme, parts of the MeSH tree, and the International Classification of Diseases, Tenth Edition. The results of the pilot study for this collection demonstrated that automation can create useful semantic metadata, but refinements are needed.

The hundreds of person-hours of work in creating an online data source of searchable bibliographic metadata from this paper collection have resulted in a low-maintenance, compact, and searchable collection of select medical literature. Today, the collection is curated by one full-time librarian and has worldwide viewings. The JMI database permits viewing of past medical terminologies, bygone concepts, faded ontologies, and narratives of watershed events as expressed in their original nineteenth and twentieth century writings.
